# Inter-rater reliability and test-retest reliability of the foot posture index (FPI-6) for assessing static foot posture in elderly female patients with knee osteoarthritis and its association with quadriceps muscle tone and stiffness

**DOI:** 10.3389/fbioe.2024.1385986

**Published:** 2024-06-21

**Authors:** XingXing Shen, Shuai Wang, Jiahao Chen, Junyi Li, Congcong Li, Ruian Xiang, Chuanxi Zhao, Xuemeng Xu

**Affiliations:** ^1^ The Fifth Clinical Medical School, Guangzhou University of Chinese Medicine, Guangzhou, China; ^2^ Guangdong Second Traditional Chinese Medicine Hospital, Guangzhou, China

**Keywords:** knee osteoarthritis, FPI-6, foot posture assessment, reliability, quadriceps, muscle tone, stiffness

## Abstract

**Objective:**

1. To assess the Inter-rater reliability and test-retest reliability of FPI-6 total score and individual scores in static foot posture evaluation among elderly female patients with knee osteoarthritis (KOA), aiming to establish the reliability of the FPI-6 scale. 2. To investigate the disparity between dominant and non-dominant quadriceps characteristics in elderly female KOA patients, as well as explore the correlation between quadriceps characteristics and abnormal foot posture, thereby offering novel insights for the prevention and treatment of KOA.

**Methods:**

The study enrolled a total of 80 lower legs of 40 participants (all female) with unilateral or bilateral KOA, who were assessed by two raters at three different time points. The inter-rater and test-retest reliability of the FPI-6 was evaluated using the intra-class correlation coefficient (ICC), while the absolute reliability of FPI-6 was examined using the standard error of measurement (SEM), minimum detectable change (MDC), and Bland-Altman analysis. The internal consistency of FPI-6 was assessed using Spearman’s correlation coefficient. Additionally, MyotonPRO was employed to assess quadriceps muscle tone and stiffness in all participants, and the association between quadriceps muscle tone/stiffness and the total score of FPI-6 was analyzed.

**Result:**

Our study found excellent inter-rater and test-retest reliability (ICC values of 0.923 and 0.931, respectively) for the FPI-6 total score, as well as good to excellent reliability (ICC values ranging from 0.680 to 0.863 and 0.739–0.883) for individual items. The SEM and MDC values for the total score of FPI-6 among our study inter-rater were 0.78 and 2.15, respectively. and the SEM and MDC values for the test-retest total score of FPI-6 were found to be 0.76 and 2.11, respectively. Furthermore, the SEM and MDC values between inter-rater and test-retest across six individual items ranged from 0.30 to 0.56 and from 0.84 to 1.56. The Bland-Altman plots and respective 95% LOA showed no evidence of systematic bias. In terms of the mechanical properties of the quadriceps on both sides, the muscle tone and stiffness of rectus femoris (RF), vastus medialis (VM), and vastus lateralis (VL) were significantly higher in the non-dominant leg compared to the dominant leg. Additionally, in the non-dominant leg, there was a significant positive correlation between the muscle tone and stiffness of VM, VL, RF and the total score of FPI-6. However, in the dominant leg, only VM’s muscle tone and stiffness showed a significant positive correlation with the total score of FPI-6.

**Conclusion:**

The reliability of the FPI-6 total score and its six individual items was good to excellent. Our findings offer a straightforward and dependable approach for researchers to assess foot posture in elderly female patients with KOA. Furthermore, we observed significantly greater quadriceps tension and stiffness in the non-dominant leg compared to the dominant leg. The FPI-6 total score exhibited a significant correlation with changes in quadriceps muscle performance among KOA patients. These observations regarding the relationship between changes in quadriceps muscle performance and foot posture in elderly female KOA patients may provide novel insights for disease prevention, treatment, and rehabilitation.

## Introduction

Knee osteoarthritis (KOA) is a prevalent chronic musculoskeletal disorder (Sharma, 2021), causing pain, functional impairment, and diminished quality of life (Du et al., 2023; Geng et al., 2023). Moreover, it is the primary cause of lower limb disability among the elderly population, particularly elderly females (Primorac et al., 2020). It is estimated that approximately 30.8 million adults in China and around 300 million individuals worldwide suffer from KOA, with a significantly higher prevalence among females compared to males (Mora et al., 2018; Reina-Mahecha et al., 2023). The incidence and prevalence of KOA are escalating due to the accelerated aging process within populations. This condition profoundly impacts physical and mental wellbeing while imposing a substantial burden on public health systems (Cui et al., 2020). Knee osteoarthritis encompasses multifactorial pathogenesis. Inflammatory processes and biomechanical alterations in surrounding muscle tissues lead to increased load-bearing capacity and heightened activity levels within the knee joint, thereby inducing KOA development (Briggs-Price et al., 2022). Etiological factors may include age-related changes, obesity, non-infectious inflammation, traumatic injuries, as well as genetic factors (Toivanen et al., 2010). Furthermore, the biomechanical modifications in both the knee joint itself and adjacent muscle tissues play crucial roles in initiating and progressing primary KOA cases (Briggs-Price et al., 2022). Additionally, abnormal foot posture may also contribute to the onset of KOA (Chen et al., 2021).

From the perspective of lower limb biomechanics, abnormal foot posture can have a significant impact on ankle joint function (Liu et al., 2023). The ankle joint serves as the pivotal axis for force transmission in the lower limb. Deviations in ankle joint position can disrupt this axis, leading to biomechanical alterations in the knee joint, hip joint, spine, and even the center of gravity responsible for human stability. Ultimately, these modifications exert an influence on joint functionality and diminish overall stability (Resende et al., 2016). Some studies suggest that excessive varus or valgus of the subtalar joint can disrupt the biomechanics of the tibiofemoral joint by increasing the angle of external rotation or internal rotation of the tibia (Gates et al., 2017). The biomechanical abnormalities in foot and ankle joints can impact the rotational and frontal measurements of the knee joint (Gates et al., 2017). Several studies have indicated that any abnormality in foot posture among patients with KOA, whether it is pronation or supination, affects force distribution throughout the entire lower limb (Al-Bayati et al., 2018). Furthermore, utilizing foot orthosis and medial arch support has been shown to reduce knee joint load in individuals with medial knee osteoarthritis (Dessery et al., 2017). Therefore, considering that changes in foot posture can lead to biomechanical disorders in lower limbs which subsequently affect normal positioning of ankle and knee joints, evaluating foot posture becomes crucial for understanding KOA pathogenesis and formulating appropriate treatment measures.

In previous studies, a number of methodologies have been employed to assess foot posture and function, including radiography techniques (Patel et al., 2020), motion analyzers (Menz et al., 2013), and MatScan systems (Taş and Çetin, 2019). Despite their proven reliability, these methods also possess several drawbacks. Radiography necessitates prolonged exposure to ionizing radiation, which may pose risks to human health (Manning et al., 2020). Moreover, both motion analyzers and MatScan systems are prohibitively expensive and entail time-consuming data acquisition processes that are not readily embraced by researchers or patients. In contrast, the foot posture index-6 (FPI-6) is regarded as a rapid, straightforward, and cost-effective measurement approach. It exhibits an accuracy level of up to 80% when compared with modern machine sensing systems for evaluation outcomes (Motantasut et al., 2019). Additionally, FPI-6 enables swift assessment of actual foot position changes in the forefoot, hindfoot, and midfoot regions; prediction of dynamic and static foot position alterations, as well as classification of foot positions into pronation, neutral position, and supination based on quantitative analysis results (Redmond et al., 2006). Therefore, FPI-6 holds significant value in assessing foot posture. However, FPI-6 has not been extensively utilized for evaluating static foot posture in KOA patients. Establishing the reliability of FPI-6 in assessing the foot posture of KOA patients is particularly important, specially among elderly females.

The quadriceps femoris (QF) is the largest muscle group in the lower limbs (Plastow et al., 2023), comprising of the rectus femoris (RF), vastus medialis (VM), vastus lateralis (VL), and vastus intermedius (VI). The QF serves as the primary extensor group and plays a crucial role in knee joint activities. Degeneration of the QF can lead to a decrease in its protective effect on the knee joint (Chen et al., 2023). Previous research has indicated that long-term asymmetric foot posture in patients with knee osteoarthritis (KOA) can result in mechanical imbalance between both lower limbs, affecting muscles near the knee joint, potentially leading to changes in QF muscle properties such as muscle tone, stiffness and elasticity (Chen et al., 2023). These alterations in muscle properties can be assessed using a non-invasive digital palpation device called MyotonPRO, which enables quick and accurate evaluation of superficial muscle characteristics (Aird et al., 2012).

The higher prevalence of KOA in women compared to men (Katz et al., 2021), along with inherent differences in lower limb muscle strength, mass, and fat content between genders, can lead to alterations in static foot posture (Chen et al., 2023). It remains unclear whether these variations impact the applicability of the FPI-6 index across all populations. Therefore, a specific investigation into the inter-rater reliability and test-retest reliability of the FPI-6 index for evaluating female foot posture is deemed necessary to establish its overall reliability. Additionally, our study aims to assess disparities in quadriceps muscle characteristics among older women with KOA and analyze their association with the FPI-6 index. The findings contribute to the quantification of symptom severity in patients with KOA, thereby establishing a novel theoretical foundation for the development of preventive and therapeutic strategies targeting KOA.

## Materials and methods

### Study design

The prospective clinical study was conducted at the orthopedic outpatient Clinic of Guangdong Second Traditional Chinese Medicine Hospital from June to December 2023. Patients with KOA were identified through the electronic medical records of Guangdong Second Traditional Chinese Medicine Hospital, in conjunction with self-reports from clinicians who diagnosed OA based on the diagnostic criteria provided below. Subsequently, a referral letter was submitted to recruit eligible subjects. All potential participants underwent a screening call and then participated in a face-to-face interview to complete informed consent and baseline assessment. The study protocol received approval from the Ethics Committee of Guangdong Second Traditional Chinese Medicine Hospital [No. 2021(K58)] and was registered with the Chinese Clinical Trial Registration Center (Registration No. ChiCTR2100050269). Prior to participation, all participants provided written informed consent and could withdraw from the study at any time.

### Participants

Zou, (2012) proposed a method for estimating the required sample size in reliability studies. This method aimed to ensure that the lower limit of the one-sided 95% confidence interval is no less than 0.7 with an 80% probability, assuming an expected value of 0.725 for the in-class correlation coefficient. According to the formula, a minimum sample size of 16 participants is needed to complete the study. However, in this study, a sample size of 40 participants was estimated. The inclusion criteria were as follows: 1) Female patients with unilateral or bilateral KOA were diagnosed by orthopedic clinicians from the Second Hospital of Traditional Chinese Medicine of Guangdong Province according to the clinical criteria established by the American College of Rheumatology; Knee pain and including at least 3 of 6: Age >50 years; Stiffness <30 min; Crepitus; Bony tenderness; Bony enlargement; No palpable warmth (Altman et al., 1986); 2)The BMI is within the range of 18–25 kg/m^2^ and the age range of the patients is between 50 and 75 years old; 3) Kellgren/Lawrence (Kellgren and Lawrence, 1957) (K/L) grade ≥1 for unilateral or bilateral knees; 4) Occurrence of predominantly medial compartment KOA; 5) Ability to maintain static standing on the platform. The exclusion criteria were: 1) Presence of other inflammatory arthritis; 2) Accompanied by neurological diseases, such as stroke, spinal related diseases, Parkinson’s disease, serious cardiovascular or respiratory diseases, or other musculoskeletal diseases; 3) Use of medications affecting muscle tone, stiffness, and other properties within the last month; 4) Congenital or traumatic deformities in the lower extremity; 5) Inability to participate the retest; 6) Vigorous exercise within 48 h prior to the study. The study exclusively enrolled patients with medial compartment KOA due to its significantly higher incidence compared to lateral compartment KOA in China. A total of 80 lower legs of 40 patients with unilateral or bilateral KOA were included based on inclusion and exclusion criteria, comprising 25 patients with unilateral pain and 15 patients with bilateral pain. Subsequently, the leg experiencing more severe symptoms or rated higher on the Visual Analog Scale (VAS) for leg pain was designated as the relatively severe leg (RSL), while the leg exhibiting mild or no symptoms was categorized as the relatively moderate leg (RML).

### Evaluation steps of foot posture

In this study, two raters (XX S and SW) independently utilized the FPI-6 to assess the static foot posture of all participants (Our research team members were assigned sequential numbers, and two members with over 5 years of clinical experience in musculoskeletal research were randomly selected as raters). Both raters underwent an 8 weeks training course on FPI-6 and interacted with each other to familiarize themselves with each item within the assessment tool, aiming to minimize inter-rater discrepancies. During the assessment period, the subjects were asked to stand barefoot in a neutral position, with bilateral support, arms naturally drooping on both sides, and eyes looking straight ahead to avoid foot posture changes caused by rotation (Chen et al., 2021). Two raters independently evaluated and scored each item of FPI-6 in a separate table. The FPI-6 score consisted of six items: (1) talar head palpation, (2) curves above and below the lateral malleolus, (3) talonavicular joint bulging, (4) caalcaneal frontal plane position, (5) medial longitudinal arch height and congruence, (6) forefoot abduction or adduction (Wang et al., 2023). Each item was assigned a score ranging from −2 to 2 points with a total score ranging from −12 to +12. When the total score of FPI-6 was ≥10, the foot was classified as a severe pronation posture, 6 to 9 was classified as a mild pronation posture, 0 to 5 was classified as a neutral posture, −1 to −4 was classified as a mild supinated posture, and ≤−5 was classified as a severe supinated posture (Redmond et al., 2006). After completion of assessment by the first evaluator (XX S), participants were required to maintain their original position while being assessed by a second evaluator (S W), who evaluated them in the same position. Inter-rater reliability was calculated using measurement data from both evaluators. One week later, the rater (XX S) conducted a retest on all participants and used the two measurements of the same rater (XX S) to calculate the test-retest reliability. Each rater assessed and scored each item independently using separate tables dedicated for this purpose. Throughout the evaluation process, neither of the raters knew the basic information about the participants and the pain site. The raters were blinded to each other and their own data.

In addition, the bilateral extremities data were calculated using the same method as (Evans et al., 2003) to determine whether the left and right limb data can be combined and analyzed. The results indicated that simultaneous analysis of both limbs’ data was feasible. Therefore, this study included both feet of participants for summary and analysis.

### Evaluation of properties of quadriceps femoris

The study utilized a non-invasive handheld machine (MyotonPRO, Estonia, serial number: 000,297, product manufacturer code: 1308600502) to assess the quadriceps performance of participants. Previous researchers have confirmed the excellent inter-rater and test-retest reliability of MyotonPRO in evaluating quadriceps tone and stiffness (Aird et al., 2012; Chen et al., 2019). Moreover, due to its inability to measure VI located deep inside the RF, we solely measured muscle tone and stiffness of the RF, VM, and VL. The members of the research team who conducted this evaluation (two randomly selected individuals from our research team, CC-L and JY-L) have received professional training by MyotonPRO and have completed professional evaluation tests. During the training, two participants actively engaged in interactive activities to familiarize themselves with the utilization program of MyotonPRO and minimized discrepancies between raters. Prior to formal data collection, we selected and marked the measurement locations: RF was measured two-thirds of the distance between the anterior superior iliac spine and the upper margin of the patella; When selecting the measurement points for VM and VI, participants were required to actively contract their lower limbs to extend the hip and knee joints, and the muscular abdominal bulge near the knee joints on both sides were the measurement points (Chen et al., 2019). The measurement sequence was as follows: left RF, right RF, left VM, right VM, left VL, right VL. The room temperature was maintained at approximately 25°C during measurements. Participants were instructed to rest for 5 minutes before the start of the test, and and then to maintain a supine position on the examination couch. Furthermore, participants were asked to hold their breath for 5 s at the end of inhalation to mitigate confounding factors arising from fluctuations in abdominal pressure during natural breathing cycles prior to conducting the test (Chen et al., 2019). After the commencement of measurement, the operator held the MyotonPRO in one hand and placed the device probe vertically on the surface of the participant’s skin at the marked position. Upon applying a specific depth of pressure to the probe, an indicator transition from red to green signified the initiation of measurement, followed by five consecutive vibrations indicating its completion. The instrument can apply a pretension pressure of 0.18N to slightly compress the subcutaneous superficial tissue and rapidly releases a mechanical impulse force of 0.58N for 15 milliseconds, inducing a naturally damped oscillation in the muscle tissue. Based on the oscillation signal, the frequency (F; Hz) and stiffness (S; N/m) of the oscillation are determined (Wu et al., 2021). During the study, the researcher did not know essential participant information and was unaware of which side was experiencing pain. Two members of the research team performed the tests on a single participant, and the average of the two measurements was taken as the final value. Following the study, the coefficient of variation (CV) was observed. If the CV exceeds 3%, it indicated that the error was too large and needed to be retested.

### Statistical analysis

We used SPSS 24.0 for Windows (IBM Corp., NY, United States) to conduct data analysis. The Shapiro-Wilks test was used to evaluate the normality of the collected data. The measurement data conforming to the normal distribution were represented by mean and standard deviation (SD), the measurement data not conforming to the normal distribution were represented by median and interquartile distance (IQR), and the qualitative variables were represented by counts and percentages. To assess the inter-rater reliability and test-retest reliability of FPI-6, we employed intra-class correlation coefficients (ICCs). ICC is calculated as follows: ICC = *σb* ^*2*^/(*σb* ^*2*^ *+σw* ^*2*^), *σb* ^*2*^ is the between-sample variance and *σw* ^*2*^ is the within-sample variance (Pleil et al., 2018). ICC values ranged from 0 to 1, with higher values indicating greater reliability (Yang et al., 2022). Inter-rater reliability was assessed using the two-way random model and average measurement method, while test-retest reliability was evaluated using the two-way mixed model and single measurement method. The ICC values were interpreted as follows:, ICC <0.4 indicated poor reliability, ICC≥ 0.4 ≤ 0.59 indicated medium reliability, ICC≥ 0.6 ≤ 0.74 indicated good reliability, and ICC≥ 0.75 indicated excellent reliability (van Geel et al., 2020). Furthermore, additional metrics including standard error (SEM), minimum detectable change (MDC), and 95% agreement limit (LOA) were calculated to assess absolute confidence (Wang et al., 2023). The measurement of SEM is associated with ICC, which can be calculated using the following formula: SEM = standard deviation (SD) ×√1-ICC. SEM is commonly utilized in clinical measurement procedures to minimize variability among samples. A lower value of SEM indicates higher accuracy in measurements. To determine the true change in fpi-6 score relative to random error, the MDC at a 95% confidence level can be calculated using the following formula: MDC = 1.96 × SEM × √2 (Weir, 2005). The 95% LOA are computed as follows: mean difference between devices ±1.96 × SD (Bland and Altman, 2007). The Blain-Altman plot from Medcalc software version 20.0 (Medcalc, Ostend, Belgium) was utilized to assess the system bias of inter-rater test reliability and test-retest reliability. Chi-square test was employed to analyze the differences in foot postural classification data. The comparison of muscle characteristics of relatively severe leg (RSL) and relatively moderate leg (RML) in the same patient was performed by paired sample t-test. The effect size is utilized to examine the magnitude of a significant difference. Cohen’s d value serves as an indicator for the extent of the effect size. A larger value indicates a greater disparity. Spearman correlation coefficient was used to evaluate the internal consistency of FPI-6 by assessing the correlation between each item and the total score, as well as between the total score of FPI-6 and muscle tension and stiffness of RSL and RML. The Spearman correlation coefficient indicates a “strong” correlation when the values are >0.7, a “good” correlation between 0.50 and 0.70, a “fair” or “moderate” correlation between 0.3 and 0.5, and any value < 0.30 represents poor correlation (Hazra and Gogtay, 2016). Consistency was considered acceptable when the spearman correlation coefficient exceeded 0.30 (Koo and Li, 2016). Statistical significance was defined as *p* < 0.05.

## Result

### Participants characteristics

**FIGURE 1:**
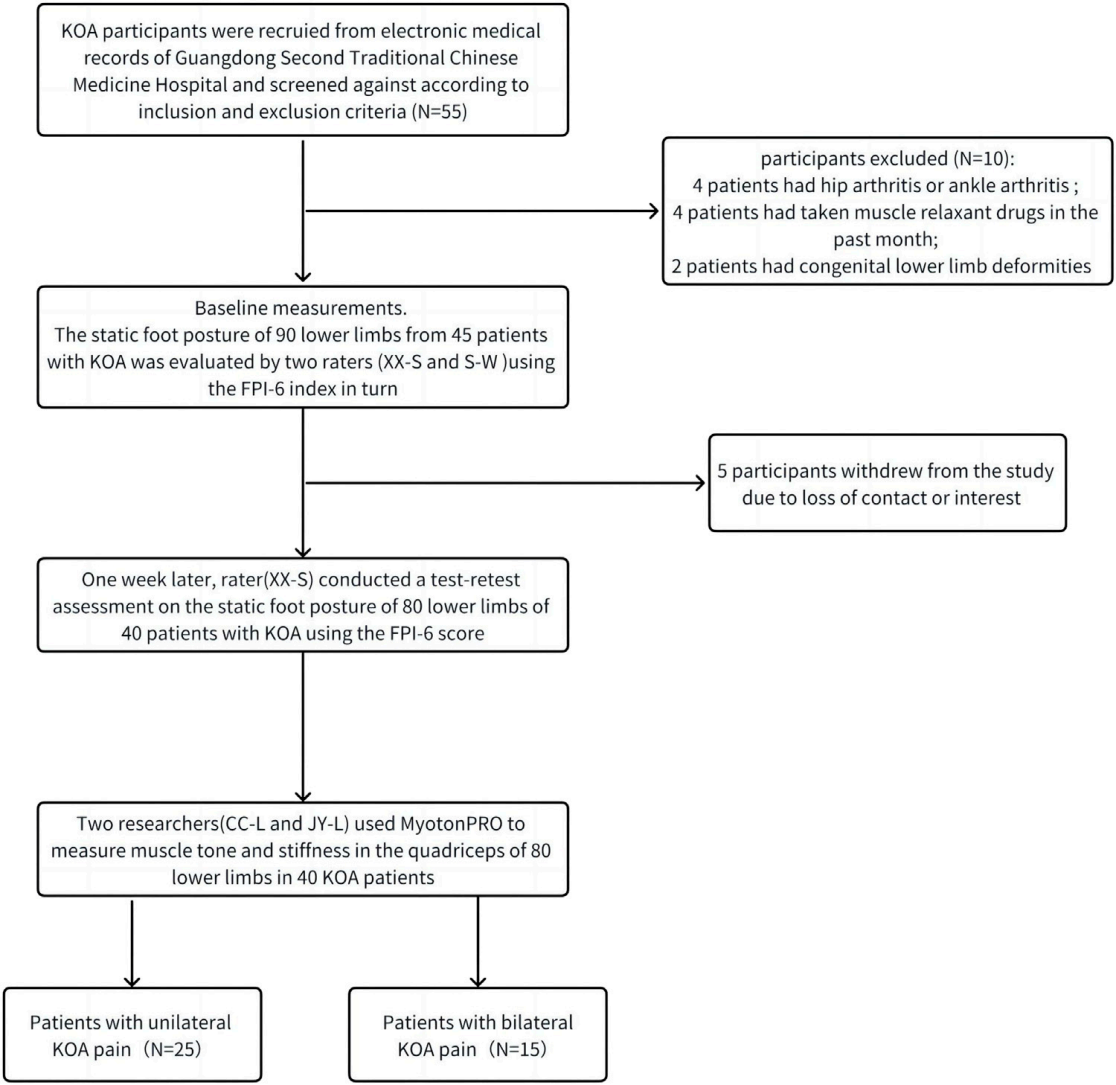
The selection and testing process of KOA participants.

Following the inclusion and exclusion criteria, a total of 80 lower legs of 40 patients with KOA were included in this study. The characteristics and foot morphology of participants were shown in Table 1 and the selection and testing process of KOA participants was shown in Figure 1.

**TABLE 1:** Participant characteristics (n = 40).

Variable	Mean ± SD or n (%)
Age (years)	65.28 ± 5.93
Height (cm)	163.03 ± 6.37
Weight (kg)	59.25 ± 7.31
BMI (kg/m2)	22.27 ± 2.19
Gender (Female/Male)	40/0
Kellgren-Lawrence classification	
Grade 1	5 (12.5%)
Grade 2	15 (37.5%)
Grade 3	17 (42.5%)
Grade 4	3 (7.5%)
Limb involvement	
Unilateral	25 (62.5%)
Bilateral	15 (37.5%)
RSL (left/right)	20/20
RML (left/right)	18/22

SD, standard deviation; BMI, body mass index; RSL, relatively severe leg; RML, relatively moderate leg.

### Inter-rater reliability

The inter-rater reliability results of FPI-6 total score in 80 limbs from 40 patients were presented in Table 2. The ICC value of FPI-6 total score was found to be 0.923 (95%CI 0.883–0.950), the SEM value of FPI-6 total score was 0.78, and the MDC value of FPI-6 total score was 2.15. The ICC values of 6 items were all >0.6, the SEM values were between 0.36 and 0.56, and the MDC values were between 1.00 and 1.56. The results shown that the FPI-6 scale has excellent inter-rater reliability, as shown in Table 2.

**TABLE 2:** Inter-rater reliability of the FPI-6.

Variable	SD	ICC(95% CI)	SEM	MDC	*p*-value
Total FPI-6	2.80	0.923 (0.883, 0.950)	0.78	2.15	<0.01
Item 1	1.02	0.695 (0.562, 0.793)	0.56	1.56	<0.01
Item 2	0.98	0.760 (0.649, 0.839)	0.48	1.33	<0.01
Item 3	0.87	0.680 (0.542, 0.782)	0.49	1.36	<0.01
Item 4	0.97	0.863 (0.794, 0.910)	0.36	1.00	<0.01
Item 5	0.86	0.752 (0.639, 0.834)	0.43	1.19	<0.01
Item 6	0.88	0.756 (0.644, 0.837)	0.43	1.20	<0.01

SD, standard deviation; SEM, standard error of measurement; MDC, the minimal detectable change at a 95% confidence level; Total FPI-6, Foot total score of FPI-6; ICC, intraclass correlation coefficient; Item 1, Talar head palpation; Item 2, Curves above and below the lateral malleolus; Item 3, Talonavicular joint bulging; Item 4, Calcaneal frontal plane position; Item 5, Medial longitudinal arch height and congruence; Item 6, Forefoot abduction or adduction.

### Test–retest reliability

The test–retest reliability results of FPI-6 total score in 80 limbs from 40 patients were presented in Table 3. The ICC value of FPI-6 total score was 0.931 (95%CI 0.893–0.956), the SEM value of FPI-6 total score was 0.76, and the MDC value of FPI-6 total score was 2.11. The ICC values of 6 items were all >0.7, the SEM values were between 0.30 and 0.49, and the MDC values were between 0.84 and 1.35. The results shown that the FPI-6 scale has excellent test–retest reliability, as shown in Table 3.

**TABLE 3:** Test–retest reliability of the FPI-6.

Variable	SD	ICC(95% CI)	SEM	MDC	*p*-value
Total FPI-6	2.90	0.931 (0.893, 0.956)	0.76	2.11	<0.01
Item 1	0.98	0.871 (0.799, 0.917)	0.35	0.98	<0.01
Item 2	0.95	0.739 (0.594, 0.833)	0.49	1.35	<0.01
Item 3	0.87	0.747 (0.606, 0.838)	0.44	1.21	<0.01
Item 4	0.96	0.831 (0.737, 0.892)	0.39	1.09	<0.01
Item 5	0.89	0.883 (0.817, 0.925)	0.30	0.84	<0.01
Item 6	0.92	0.851 (0.768, 0.905)	0.36	0.98	<0.01

SD, standard deviation; SEM, standard error of measurement; MDC, the minimal detectable change at a 95% confidence level; Total FPI-6, Foot total score of FPI-6; ICC, intraclass correlation coefficient; Item 1, Talar head palpation; Item 2, Curves above and below the lateral malleolus; Item 3, Talonavicular joint bulging; Item 4, Calcaneal frontal plane position; Item 5, Medial longitudinal arch height and congruence; Item 6, Forefoot abduction or adduction.

### The level of agreement

**FIGURE 2:**
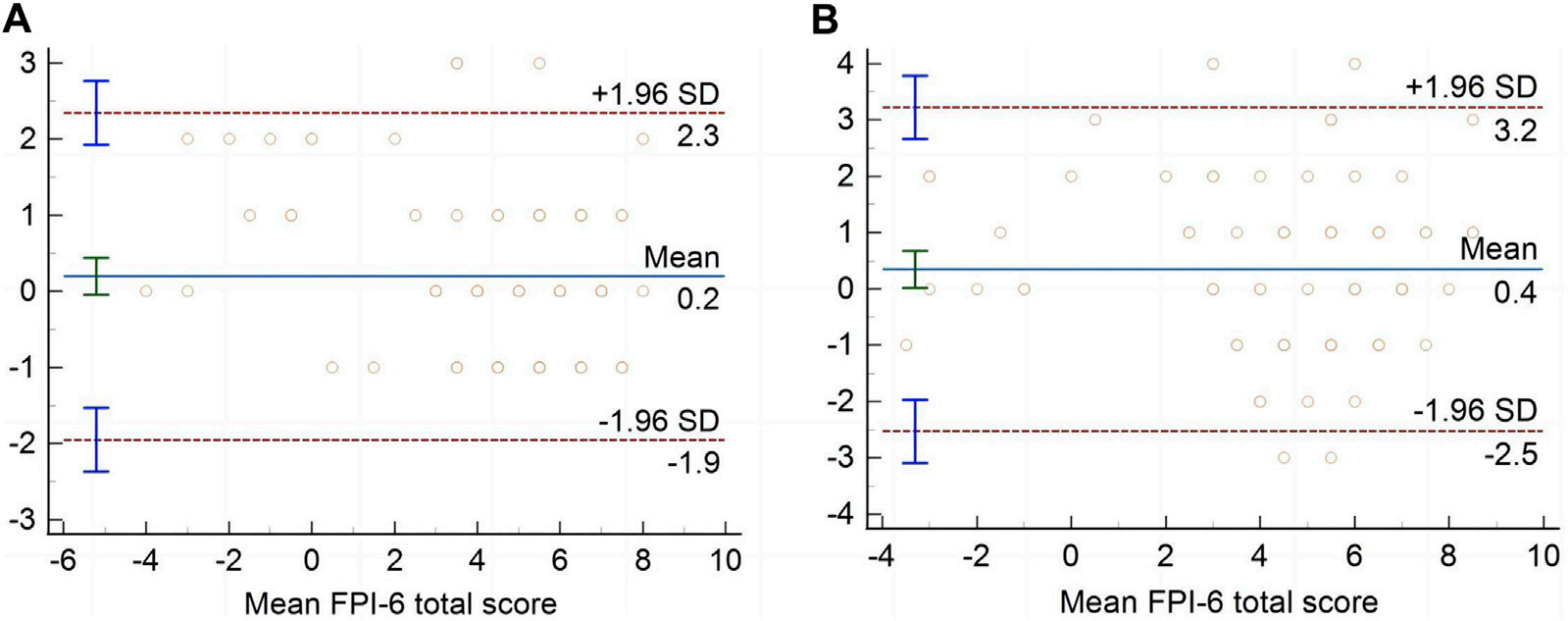
Bland–Altman plots of FPI-6 total score for inter-rater **(A)** and test–retest **(B)**.

The Bland-Altman graphs with the mean difference and 95% LOA of the consistency level were shown in Figure 2. The average difference of the total score of FPI-6 between inter-rater and test-retest was 0.2 and 0.4, and the lower limit and upper limit were −1.9–2.3 and −2.5–3.2, respectively. The results indicated that there was little systematic bias, and the acceptable agreement of FPI-6 total scores between the inter-rater and the test-retest was excellent.

### Correlation analysis

The correlation between each item and the total score of FPI-6 was shown in Table 4. The results showed that the Spearman correlation coefficients of all 6 items were >0.3 (*p* < 0.01). Therefore, there was a statistically significant positive correlation between each item and FPI-6 total score.

**TABLE 4:** The correlations between each item and FPI-6 total score.

Variable	Spearman’s correlation coefficient	*p*-value
Item 1	0.537	<0.01
Item 2	0.397	<0.01
Item 3	0.353	<0.01
Item 4	0.454	<0.01
Item 5	0.505	<0.01
Item 6	0.414	<0.01

Item 1, Talar head palpation; Item 2, Curves above and below the lateral malleolus; Item 3, Talonavicular joint bulging; Item 4, Calcaneal frontal plane position; Item 5, Medial longitudinal arch height and congruence; Item 6, Forefoot abduction or adduction.

### Classification of the static foot posture

The static foot posture classification at the three assessment times was presented in Table 5. A total of 80 limbs from 40 participants were evaluated at three different time points. During the first assessment conducted by rater 1 (XX S), it was observed that out of the assessed feet, 32 were classified as pronated, 10 as supinated, and 38 as normal. None of the feet were categorized as highly pronated or highly supinated. In the second assessment performed by rater 2 (S W), it was found that among the evaluated feet, 31 were classified as pronated, 5 as supinated, and 44 as normal and none of the feet fell into the category of highly pronated or highly supinated. One week later, during the third assessment carried out by rater 3 (XX S), The results indicated that 1 foot was highly pronated, 33 feet were pronated, 8 feet were supinated, and 38 feet were normal. None of the feet was classified as highly outturned. There was no significant difference in classification of foot posture for inter-rater (*χ2* = 2.12, *p* = 0.35) and test–retest (*χ2* = 1.24, *p* = 0.74).

**TABLE 5:** Classification of the static foot posture in the three moments of assessment.

Rater	Highly pronated	Pronated	Normal	Supinated	Highly supinated	Total	χ2 value	*p*-value
1	0	32	38	10	0	80		
2	0	31	44	5	0	80	2.12^a^	0.35^a^
3	1	33	38	8	0	80	1.24^b^	0.74^b^

1, rater (XX S); 2, rater (S W); 3, rater (XX S) approximately 1 week later; a, inter-rater reliability; b, test–retest reliability;

### The muscle tone and stiffness of the quadriceps

The results showed that the muscle tone of RF, VI and VL in RML was higher than those on RSL (*p* < 0.05), and the muscle tone of VM was significantly increased (*p* < 0.001). Additionally, the muscle stiffness of RF, VI and VL in RML was higher than those on RSL (*p* < 0.05), and the muscle stiffness of VL increased significantly (*p* < 0.001), as shown in Table 6.

**TABLE 6:** Difference between muscle tone and quadriceps between both sides (n = 40).

Muscle	Variable	RML	RSL	*MD*	*T* value	*p*-value	Cohen’s d value	Statistical power
RF	Muscle tone (Hz)	13.74 ± 1.67	14.37 ± 1.29	−0.63	−2.210	0.033	0.349	0.926
	Stiffness (N/m)	251.75 ± 27.14	263.63 ± 25.84	−11.88	−2.319	0.026	0.367	0.946
VM	Muscle tone (Hz)	13.34 ± 1.29	14.08 ± 1.16	−0.74	−3.731	<0.001	0.590	0.999
	Stiffness (N/m)	239.78 ± 18.63	249.08 ± 27.28	−9.30	−2.369	0.023	0.375	0.954
VL	Muscle tone (Hz)	14.51 ± 1.84	15.06 ± 1.90	−0.55	−2.257	0.030	0.357	0.936
	Stiffness (N/m)	266.13 ± 28.04	282.78 ± 30.19	−16.65	−3.448	<0.001	0.545	0.999

RSL: relatively severe leg, RML: relatively moderate leg; RF: rectus femoris; VM: vastus medialis; VL: vastus lateralis; MD: mean difference.

### The relationship between FPI-6, total score and quadriceps muscle performance

The final result was determined by selecting the average value of FPI-6 total score at three time points, followed by conducting correlation analysis between muscle tone and stiffness of the quadriceps muscle. The findings revealed a statistically significant positive correlation between VM muscle tone, stiffness, and FPI-6 total score in RML (ρ > 0.3, *p* < 0.05), However, no significant correlation was observed between RF and VI muscle tone, stiffness, and FPI-6 total score in RML (*p* > 0.05). Furthermore, there was a significantly positive correlation between the muscle tone and stiffness of RF, VI, and VM in RSL with the FPI-6 total score (ρ > 0.3, *p* < 0.05), as presented in Table 7.

**TABLE 7:** The relationship between FPI-6 total score and the muscle tone and stiffness of the quadriceps muscle.

Muscle	Variable	RML		RSL	
		Spearman’s ρ	*P* -value	Spearman’s ρ	*P* -value
RF	muscle tone (Hz)	0.197	0.223	0.491	<0.001
	stiffness (N/m)	0.300	0.060	0.591	<0.001
VM	muscle tone (Hz)	0.762	<0.001	0.686	<0.001
	stiffness (N/m)	0.536	<0.001	0.485	0.002
VL	muscle tone (Hz)	0.239	0.138	0.698	<0.001
	stiffness (N/m)	0.254	0.114	0.421	0.007

Spearman’s ρ:Spearman correlation coefficients, RSL: relatively severe leg, RML: relatively moderate leg; RF: rectus femoris; VM: vastus medialis; VL: vastus lateralis.

## Discussion

### Reliability of the FPI-6 index

This study demonstrated the reliability of FPI-6 in assessing static foot posture in elderly female KOA patients for the first time. The results showed that the FPI-6 total score and 6 individual items had good inter-rater reliability and test-retest reliability in assessing static foot posture in elderly female KOA patients, and there was a significant positive correlation between each individual item and the FPI-6 total score.

Regarding the reliability of the FPI-6, our study demonstrated excellent inter-rater reliability and test-retest reliability for the FPI-6 total score (ICC values were 0.923 and 0.931, respectively). Additionally, The inter-rater reliability and test-retest reliability of individual items on the FPI-6 ranged from good to excellent (ICC values ranged from 0.680 to 0.863 and 0.739 to 0.883, respectively). Notably, a recent study using the FPI-6 to assess static foot posture in individuals with low back pain reported even higher levels of inter-rater reliability for both the total score (ICC = 0.97) and each item’s inter-operator reliability (>0.75), surpassing those observed in our investigation (Yang et al., 2022). We hypothesized that the disparity in participant characteristics may explain the low ICC value observed in our study. Specifically, our study exclusively included female participants, while their study encompassed both male and female participants. The second factor contributing to this discrepancy could be attributed to varying levels of familiarity with the FPI-6 index among the two raters (Cornwall et al., 2008; Aquino et al., 2018). Regarding test-retest reliability, previous studies examining the static foot posture index using the FPI-6 index in adults and seniors have reported fair to good reliability for adults and unreliable to moderate reliability for seniors (Aquino et al., 2018). One possible reason why we observed higher test-retest reliability might be due to our raters’ enhanced understanding of the FPI-6 index (Yang et al., 2022). It has been demonstrated that different levels of comprehension regarding FPI-6 can lead to inconsistent test-retest reliability. Furthermore, inconsistent measurement intervals could also contribute to variations in test-retest reliability (Terada et al., 2014).

Moreover, the SEM and MDC values were utilized to assess absolute confidence in this study. The SEM value was employed to describe the degree of consistency or variation among the sample means, while the MDC value represented the minimum amount of actual change that could be interpreted (Furlan and Sterr, 2018). Generally speaking, a higher ICC value corresponds to smaller SEM and MDC values, indicating greater absolute reliability (Chen et al., 2019). The SEM and MDC values of the FPI-6 total score for inter-rater were 0.78 and 2.15, respectively, and the SEM and MDC values of the FPI-6 total score for test-retest were 0.76 and 2.11. Furthermore, when examining individual items within both inter-rater and test-retest analyses, SEM values ranged from 0.30 to 0.56 and MDC values ranged from 0.84 to 1.56 for all six items assessed by FPI-6 scale. In addition, Bland-Altman plots were employed in order to analyze differences between two measurements and identify any potential systematic biases present in our data set. The results indicated no significant systematic error within participant mean difference distribution patterns, and the scatter points of the values were basically within the 95%LOA range. Our findings were consistent with a previous study utilizing FPI-6 index for assessing static foot posture in patients with low back pain. Additionally, The Small SEM and MDC values and Bland-Altman diagram distribution characteristics were confirmed the reliability of the measured results in our research.

We employed the Spearman correlation coefficient to examine the association between each item and the FPI-6 total score, thereby assessing the internal consistency of the FPI-6 index. Our findings revealed a statistically significant positive correlation between each item and the total score of FPI-6 (the coefficients of 6 items were all greater than 0.3 and *p* < 0.01). These results aligned with a previous study (Terada et al., 2014), affirming excellent internal consistency of the FPI-6 index in evaluating static foot posture among elderly female patients with KOA.

Varus limb alignment is the predominant manifestation in patients with medial knee osteoarthritis, and the severity of varus limb alignment may influence the progression and incidence of KOA (Sharma et al., 2010). Previous studies have reported that individuals with medial compartment KOA exhibit a more pronated foot posture and less flexible foot movement patterns compared to controls (Zhang et al., 2017). Based on these reasons, we examined changes in foot posture across a total of 80 limbs in 40 participants. The results indicated no significant difference in foot posture between inter-rater and test-retest assessments (*p* > 0.05). In this study, out of the total 80 limbs assessed, seven feet showed changes in their postural classifications between inter-rater and test-retest evaluations, with most affected feet being classified as pronated. This result was similar to a previous study that assessed inter-rater and test-retest differences across 60 limbs and showed changes in four foot posture classifications (Wang et al., 2023), Overall, our results suggest that the FPI-6 foot posture index is clinically reliable.

### Performance difference of the QF and correlation with FPI-6

Patients with KOA commonly experience quadriceps muscle atrophy, leading to reduced knee stability and increased cartilage wear, ultimately contributing to the development of KOA (Krishnasamy et al., 2018). Our previous studies have demonstrated that KOA patients exhibit not only quadriceps atrophy but also alterations in mechanical properties (Chen et al., 2023). However, there is currently a dearth of reference datasets on quadriceps muscle performance (such as muscle tone and stiffness) in elderly female patients with knee osteoarthritis. Furthermore, we recognized that KOA can impact both quadriceps muscle performance and foot posture, Therefore, investigating the correlation between these two variables may offer novel insights into the clinical pathogenesis and treatment strategies for KOA. In this study, we employed MyotonPRO (a noninvasive muscle palpation instrument) to evaluate quadriceps muscle tone and stiffness in 40 elderly female patients with unilateral and bilateral KOA. Additionally, we analyzed the relationship between quadriceps muscle tone/stiffness and the total score obtained from FPI-6.

Muscle tone refers to the mechanical tension generated when the muscle is in a state of complete relaxation. Abnormally elevated muscle tension can result in vasoconstriction, impeding blood supply and exacerbating muscle strain (Schoenrock et al., 2018; Wu et al., 2021). Stiffness reflects the capacity of muscles to resist contraction or mechanical forces that induce muscular deformation (Kocur et al., 2019). Increased stiffness leads to diminished muscle function. Currently, the evaluation of muscle tone and stiffness often relies on imperfect methods such as the Ashworth scale or durometer, which suffer from issues like inadequate accuracy (Van Deun et al., 2018) and inconvenient operation (Dellalana et al., 2019). MyotonPRO represents a novel non-invasive instrument for assessing muscle mechanical properties through palpation, offering advantages including high accuracy, affordability and easy operation (Feng et al., 2018). Previous studies have demonstrated excellent reliability of MyotonPRO in evaluating quadriceps femoris muscle tone and stiffness among healthy individuals (Chen et al., 2019), skin hardness in females (Rosicka et al., 2021), and skeletal muscle elasticity (Li et al., 2022) in healthy people. Building upon these prior investigations, our study employed MyotonPRO to assess quadriceps muscle tone and stiffness among elderly female patients with knee osteoarthritis.

The results indicated a significant increase in muscle tone and stiffness of VM, VL and RF on the non-dominant side leg compared to the dominant side leg. Our finding was similar to a previous study that utilized MyotonPRO to examine quadriceps stiffness differences between individuals with KOA and healthy controls, which found significantly higher quadriceps stiffness in KOA patients than healthy individuals (Chang et al., 2021). We propose that there are multiple factors contributing to the significantly higher muscle tone and stiffness observed on the non-dominant side compared to the dominant side. Age is one such factor that contributes to alterations in quadriceps performance. Research has demonstrated age-related declines in skeletal muscle quality and strength, potentially due to adjustments in muscle fiber type, the change of muscle fiber quantity and quality (Cvetko et al., 2012), the decrease of muscle cross-sectional area (Peng et al., 2022), and the structural degeneration of connective tissue (Kragstrup et al., 2011). Sarcopenia occurs when the mass of skeletal muscle is reduced to a certain extent, which affects muscle performance (Wu et al., 2021). Additionally, overweight or obesity may also be a significant factor in muscle performance changes (Georgiev and Angelov, 2019). The knee joints of individuals with excess weight not only experience mechanical pressure that surpasses the physiological capacity of weight-bearing knee joints but also induce alterations in the body’s metabolic pattern and body fluid structure, resulting in elevated levels of adipocytokines and related pro-inflammatory responses. They may lead to changes in quadriceps tension and stiffness (Kulkarni et al., 2016). Additionally, unhealthy lifestyle habits are also a contributing factor to modifications in quadriceps performance. Most KOA patients exhibit pain symptoms during the early and middle stages, which often leads to reduced activity in the affected limb. However, prolonged physical inactivity and non-use of the “diseased” limb can cause atrophy of the quadriceps muscle, leading to increased pain symptoms and decreased muscle performance, forming a vicious cycle (Ikemoto-Uezumi et al., 2017).

We conducted a comprehensive analysis of the correlation between quadriceps femoris tone and stiffness, as well as the total score of FPI-6. Our findings revealed that in the non-dominant leg, there was a significant positive correlation between the muscle tone and stiffness of VM, VL, RF and the total score of FPI-6. However, in the dominant leg, only VM’s muscle tone and stiffness showed a significant positive correlation with the total score of FPI-6. The pain symptoms in patients with KOA primarily localize to the knee joint itself and the surrounding tibial plateau, while exhibiting relatively mild pain manifestations in the quadriceps. The quadriceps predominantly manifests as reduced muscle mass and weakness. The observed muscle mass and weakness in the non-dominant quadriceps may indicate muscle fiber atrophy (Wen et al., 2018) or a combination of muscle edema, inflammation or lipid accumulation, and fiber atrophy (Alfuraih et al., 2019). We noted that several previous human studies have shown that excessive mechanical load on one limb may be the main cause of muscle atrophy (Tsukada et al., 2020). In unilateral lower extremity loading studies, disuse atrophy was observed in contralateral lower extremity muscles, which exhibited a significantly positive correlation with disease duration (Al-Khlaifat et al., 2016). in the dominant leg, VM is the latest developing muscle and the most vulnerable muscle in the quadriceps. in cases where one quadriceps muscle is affected by factors such as injury or surgery, VM becomes more susceptible to disuse atrophy (Chen et al., 2023).

Quadriceps weakness can result in knee instability and excessive extension. Knee hyperextension can cause anterior bending of the torso, resulting in the gravity line passing through the front of the knee, thereby forcing it into a passive hyperextended position and generating additional posterior knee torque. To compensate for strength deficiency and maintain balance, compensatory adjustments occur in both the human knee and ankle joints to preserve body equilibrium (Lewek et al., 2004; Øiestad et al., 2022). As a consequence of alterations in the vertical alignment of lower limbs in patients with KOA, abnormal stress is imposed on lower limb muscle activity (Krackow et al., 2011). The occurrence of pes varus is attributed to strain or spasm in the medial foot muscles and weakness in the anterior tibial muscle (Deu et al., 2022). When the valgus muscle group such as the peroneal muscle group, contracts, it leads to pes valgus (Lotito et al., 2011). Furthermore, Previous studies have been used to confirm that quadriceps weakness impacts gait patterns in female patients with KOA, resulting in decreased walking speed, reduced swing time, and increased support time (Spinoso et al., 2018). Changes in the mechanical properties of the quadriceps muscle, such as increased tone and stiffness, can exacerbate muscle weakness and atrophy in KOA patients, thereby further aggravating pain and gait abnormalities experienced by these patients. The abnormal foot posture exacerbates the excessive load on the knee joint, leading to prolonged contraction of the quadriceps muscle and subsequent decline in its mechanical properties (Chen et al., 2019), forming a vicious circle. Therefore, we indicated that as disease duration increases in patients with knee osteoarthritis, there would be a progressive deformation of the affected limb’s foot posture along with deteriorating mechanical properties of the quadriceps muscle, and there was a significant correlation between the two variables.

In summary, this study confirms the interpretive and retest reliability of both the total FPI-6 index and individual scores in assessing foot posture in elderly women with KOA. Furthermore, it reveals that the non-dominant leg exhibits significantly greater quadriceps tension and stiffness compared to the dominant leg. Additionally, a significant correlation is observed between changes in quadriceps function and total FPI-6 scores among KOA patients. These findings facilitate the quantitative assessment of quadriceps injury in patients with KOA and provide guidance for appropriate rehabilitation training, such as isometric and isotonic quadriceps training. These training contribute to improving the mechanical properties of the affected limb’s quadriceps and restoring normal lower limb alignment. This approach may serve as a potential strategy to correct asymmetrical foot posture and potentially benefit KOA patients.

Our current study has several limitations. Firstly, the sample size was relatively small (40 patients with a total of 80 lower limbs) and was limited to female patients with KOA. It remains unclear whether there is a correlation between FPI-6 and quadriceps muscle performance in healthy individuals. Therefore, caution should be exercised when applying these results to male patients with KOA or healthy individuals. In future studies, our research team intends to expand the sample size and comprehensively analyze the reliability of FPI-6 in both male KOA patients and healthy populations. Secondly, due to medial compartment osteoarthritis being predominant in China, this study only included patients primarily affected by medial KOA, which may limit the generalizability of our findings. Future studies will include patients with different types of KOA and explore the reliability of the FPI-6 index in evaluating static foot posture across various subtypes of KOA. Lastly, due to each subitem of FPI-6 index having only five possible scores for all foot types, some non-extreme foot types may not have an appropriate standard score selection available. In future, we plan to combine the FPI-6 index with gait analysis tools for a more detailed assessment of foot posture among KOA patients in order to enhance result reliability.

## Conclusion

The reliability of the FPI-6 total score and its six individual items was good to excellent. Our findings offer a straightforward and dependable approach for researchers to assess static foot posture in elderly female patients with KOA. Furthermore, we observed significantly greater quadriceps tension and stiffness in the non-dominant leg compared to the dominant leg. The FPI-6 total score exhibited a significant correlation with changes in quadriceps muscle performance among KOA patients. These observations regarding the relationship between changes in quadriceps muscle performance and static foot posture in elderly female KOA patients may provide novel insights for disease prevention, treatment, and rehabilitation.

## Data Availability

The original contributions presented in the study are included in the article/supplementary material, further inquiries can be directed to the corresponding author.
